# Global trends of research on tuberculous pleurisy over the past 15 years: A bibliometric analysis

**DOI:** 10.3389/fcimb.2022.937811

**Published:** 2022-08-30

**Authors:** Yiding Bian, Mingming Deng, Qin Zhang, Gang Hou

**Affiliations:** ^1^ Department of Pulmonary and Critical Care Medicine, Center of Respiratory Medicine, China-Japan Friendship Hospital, Beijing, China; ^2^ Graduate School of Peking Union Medical College, Chinese Academy of Medical Sciences, Peking Union Medical College, Beijing, China; ^3^ National Center for Respiratory Medicine, Beijing, China; ^4^ Institute of Respiratory Medicine, Chinese Academy of Medical Sciences, Beijing, China; ^5^ National Clinical Research Center for Respiratory Diseases, Beijing, China; ^6^ Department of Pulmonary and Critical Care Medicine, First Hospital of China Medical University, Shenyang, China

**Keywords:** bibliometrics, tuberculous pleurisy, bibliometrix, VOSviewer, diagnosis

## Abstract

Tuberculous pleurisy (TP) is a common type of extrapulmonary tuberculosis (EPTB). With the development of research and changes in TP patient characteristics, an increasing number of studies have revealed the prevalence, risk factors, and novel diagnosis techniques. Thus, this bibliometric analysis was performed to identify global scientific output characteristics and research hotspots and frontiers for TP over the past 15 years. We searched the Web of Science Core Collection (WoSCC) Science Citation Index Expanded (SCI-expanded) for literature published between 2007 and 2021 and recorded their information. The Bibliometrix software package was used for bibliometric indicator analysis, and VOSviewer was used to visualize the trends of and hotspots in TP research. A total of 1,464 original articles were reviewed, and the results indicated that the annual number of publications (Np) focusing on TP has increased over the past 15 years. China had the largest number of papers and the highest H-index, and the United States ranked first for number of citations (Nc). EGYPTIAN KNOWLEDGE BANK and PLOS ONE were the most prolific unit and journal, respectively. The use of the Xpert assay and immune-related biomarker detection to diagnose TP appears to be a recent research hotspot. This bibliometric study demonstrated that the number of publications related to TP have tended to increase. China is a major producer, and the United States is an influential country in this field. Research in the past 15 years has been predominantly clinical research. The diagnosis of TP was the focus of research, and the exploration of novel diagnostic techniques, verification of diagnostic markers, and combination of diagnostic methods have been recent research hotspots. Immune-related biomarkers should be given more attention in the field of TP diagnosis.

## Introduction

Tuberculous pleurisy (TP) is a common clinical pleural disease and one of the most frequent causes of pleural exudates globally; it is also a common manifestation of extrapulmonary tuberculosis (EPTB). The proportion of TP among all EPTB was 50.15% based on a large-scale multicenter observational study (Kang et al., 2020). In addition, TP is one of the leading causes of diagnosed pleural effusion; in India, 30-80% of plural effusions have been attributed to TP (Udwadia and Sen, 2010; Antonangelo et al., 2019). The continuous progression of TP causes a patient’s lung function to decline and can even cause the development of tuberculous empyema (Richter et al., 1994; Baumann et al., 2007; Light, 2010). A higher proportion of multidrug-resistant (MDR) tuberculosis (TB) was observed among patients with EPTB than among patients with pulmonary TB. We observed a large increase in the number of MDR TB cases, from 17.3% to 35.7%, among pleural TB cases (Pang et al., 2019). Consequently, particularly in areas where TB is endemic, TP imposes a substantial burden on patients. The antituberculosis treatment cycle is long and complex and consumes many medical resources (Chakaya et al., 2021). In the past fifteen years, research on the epidemiology, diagnostics, pathology, pathophysiology, and therapeutics of TP has greatly progressed. In addition, the advancement of diagnostic techniques and treatments has improved detection of the pathogenesis and recognition of TP, such as the differentiation of malignant pleural effusion (Huang et al., 2021; Roofchayee et al., 2021; Zeng et al., 2022), and treatment regimens, including the use of infliximab (Sousa et al., 2019) and adalimumab (Nagafuchi et al., 2013). Thus, it is necessary to publish comprehensive reports that can help scientists attain insightful knowledge and disclose research trends in the TP research field.

Bibliometric analysis is a new scientific method used to assess contributions to research fields, including contributions from countries, institutions, authors, and journals. In addition, bibliometric analysis can forecast hotspots and trends in certain research topics through information visualization (Cancino et al., 2017; Devos and Ménard, 2020; He et al., 2020; Huang et al., 2020; Wang et al., 2020). However, there is currently no bibliometric analysis of TP research. In this study, we conducted a comprehensive bibliometric analysis of TP research from 2007 to 2021 to verify TP research trends and hotspots. We hope that this research will provide a new perspective and foundation for future TP research.

## Materials and methods

### Data sources and search strategies

All data were retrieved from the Web of Science Core Collection (WoSCC) in this study. Considering rapid database updating, literature retrieval was conducted on a single day (January 1st, 2022) to avoid deviations. The publication period in this study was limited to 2007 to 2021. The search terms were as follows: TS = [tuberculosis OR (Mycobacterium tuberculosis)] AND TS = [pleurisy OR pleuritis OR (pleural effusion) OR (pleural fluid)]. Of the various publication types, only original articles and reviews published in English were included.

### Data collection and cleaning

In the present study, comprehensive publishing characteristics were extracted from the database, including the numbers of papers and citations, H-index, publication year, country/region, affiliations, authors, journal, references, and keywords. Subsequently, duplicate authors and spelling errors were deleted. Although inaccuracies in the analysis may not be completely avoided due to different versions of cited references, the same abbreviated name for different authors, and the different formats of cited journals, we believe that most of the original data are reliable. Before data analysis by VOSviewer v.1.6.10.0 (Science and Technology Research Center, Leiden University, Leiden, the Netherlands), some repetitive words were merged into one word, spelling errors were corrected, and irrelevant words were deleted. Finally, cleaned data were imported into the Bibliometrix package and VOSviewer for bibliometric analysis.

### Bibliometric analysis

Productivity is generally measured by the number of publications (Np), and the impact is measured by the number of citations (Nc), except for self-citation; these are the two main metrics for evaluating the level of research. Recently, the H-index has been increasingly used to evaluate researchers’ academic contributions and predict their future scientific achievements. The H-index combines productivity and impact by establishing a threshold connecting the Np and Nc. In other words, if a researcher has published H papers and each paper has been cited at least H times, then she or he will have an H-index. While the H-index was originally developed to assess individual academic achievement, it can be extended to describe the publication output of a country or region, institution, or journal. In addition, the impact factor (IF) obtained from the latest edition of Journal Citation Reports (JCR) has been widely regarded as one of the leading indicators of the quality and impact of medical journals. As an important indicator of contribution, the local citation score (LCS) is considered to be the Nc of an article within a specific field. To some extent, it can indicate how innovative an article is in a particular field of knowledge (Hao et al., 2019). The online bibliometric analysis platform and Microsoft Excel 2016 were used to assess the impact of authors, institutions, and journals. In addition, a bibliometric map was constructed with VOSviewer software to obtain more comprehensive outcome information based on co-occurrence. A cocitation was defined as two projects referenced by a third project. The co-occurrence of keywords measured the most frequently occurring keywords in the same document. The analysis of cocited references and co-occurring keywords can illustrate TP research hotspots.

## Results

### Overview of publications on tuberculosis pleurisy

**Figure 1:**
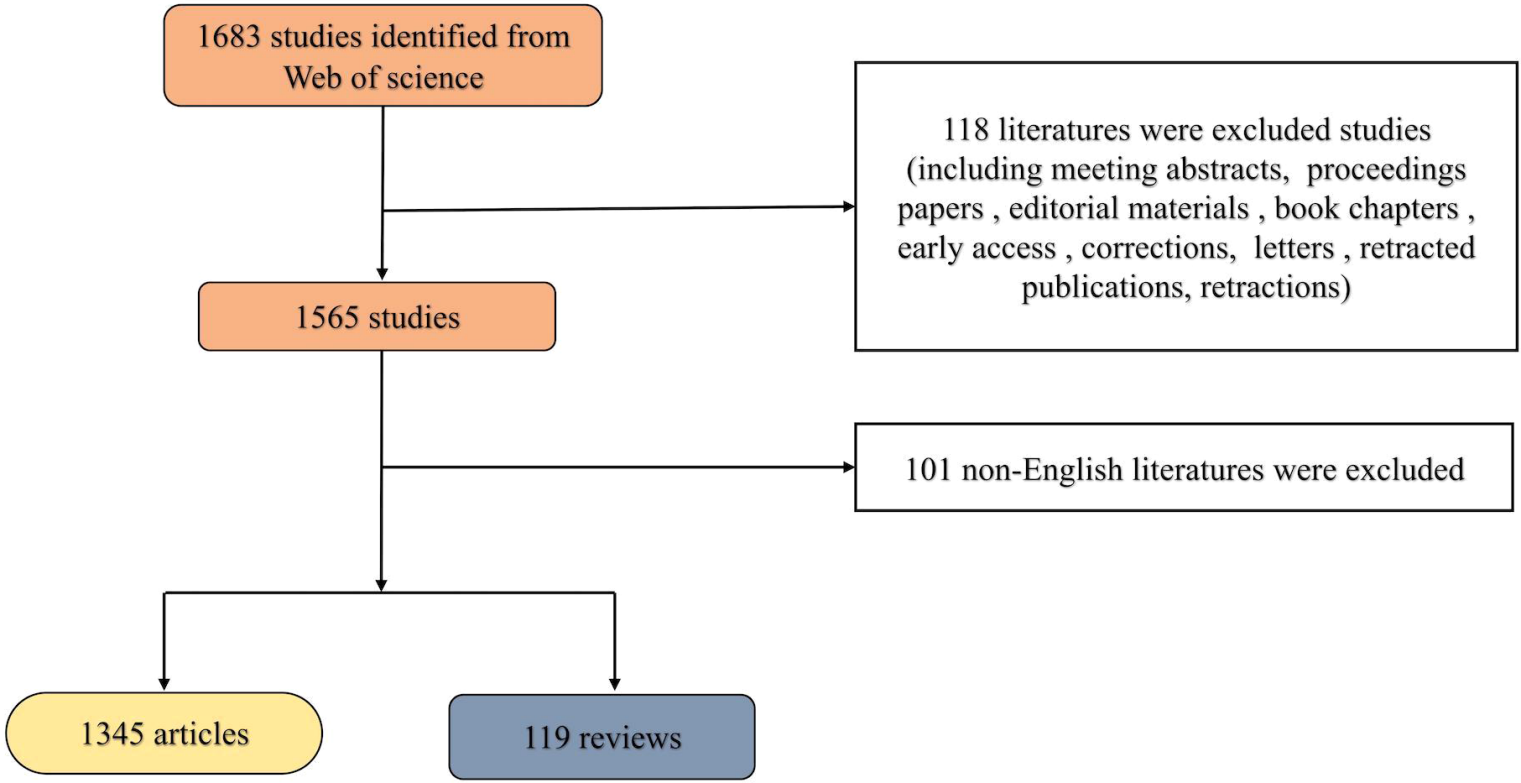
Flowchart of the screening process.

Based on the search strategy, 1464 articles published in the past 15 years were finally analyzed in our study. The detailed screening procedure is shown in **Figure 1**. The total Nc of the retrieved articles was 14,789, and the average Nc per article was 12.8. The H-index for all publications was 58.

### Annual trend of research publication quantity

**Figure 2:**
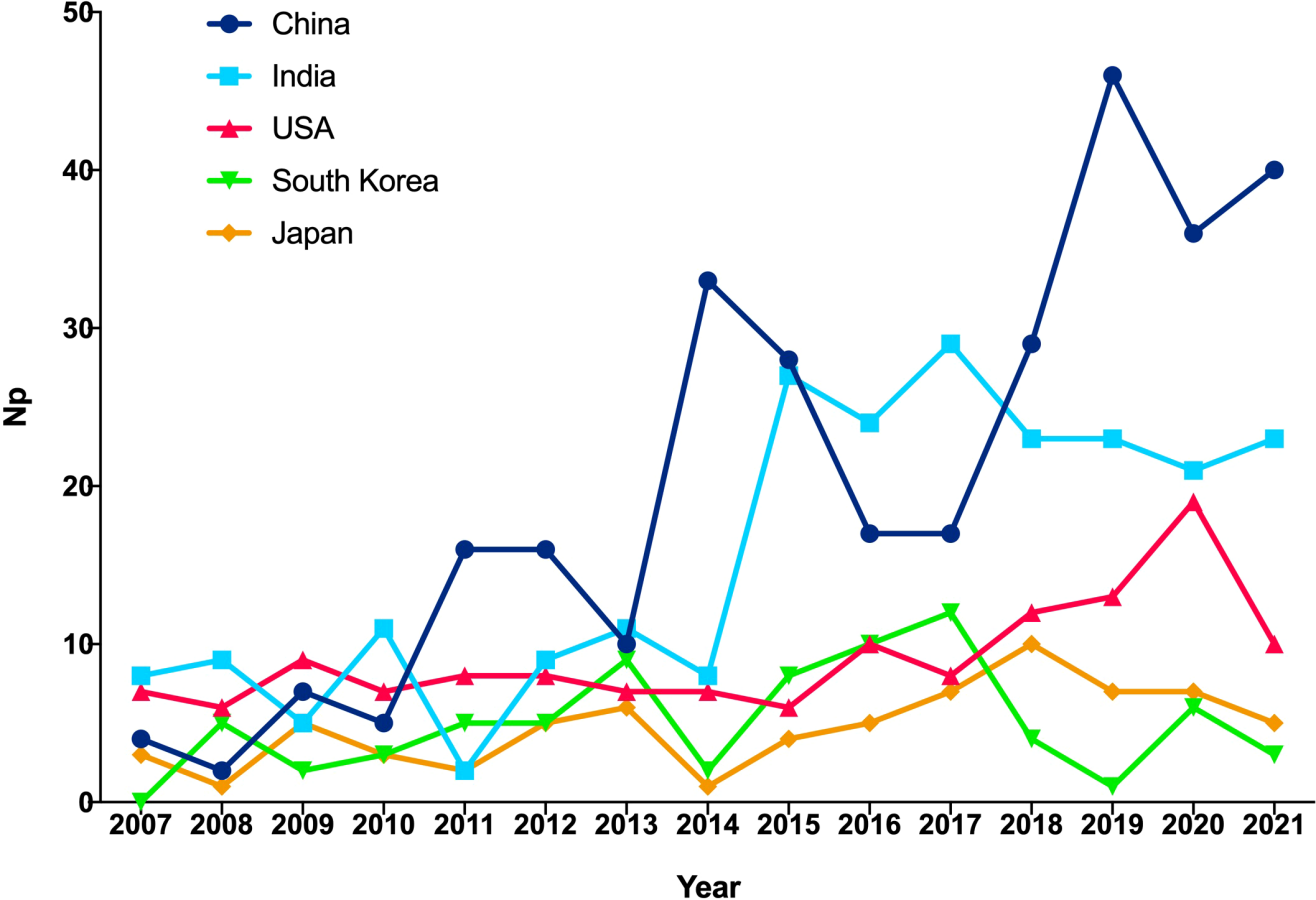
The number of publications by year over the past 10 years.

**Figure 3:**
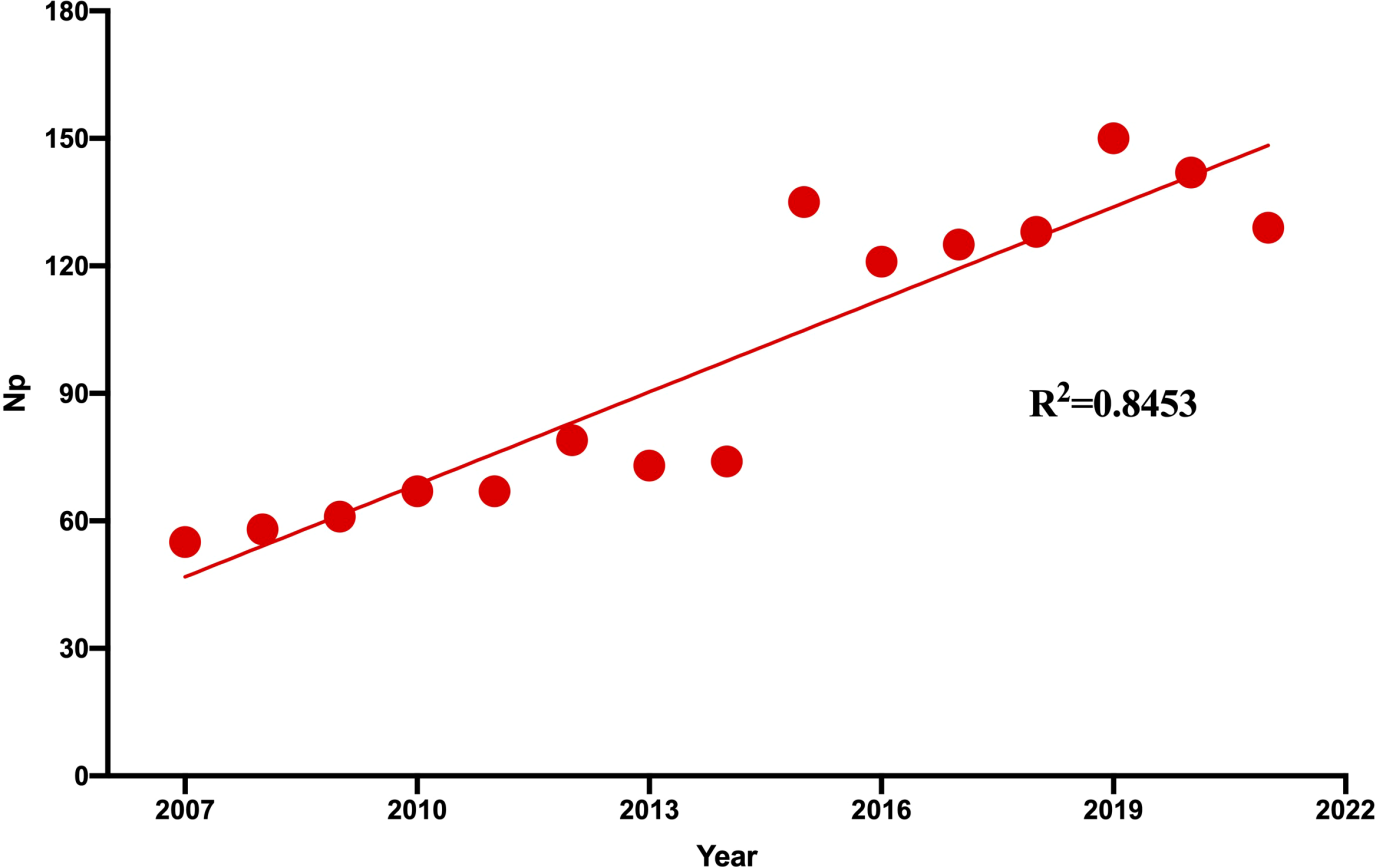
Curve fitting of the of the total annual growth trend of publications (R2 = 0.8453).

**Figure 2** shows the annual Np associated with TP. Overall, despite slight fluctuations in the past 15 years, the number of annual papers has risen from 55 in 2007 to 128 in 2021, with a peak Np in 2019. Compared with those from the United States and Japan, the annual Np from China and India increased rapidly. **Figure 3** illustrates a polynomial fit curve for the annual trend of the Np published. The annual Np was significantly correlated with publication year. As shown in **Figure 3**, the correlation coefficient R^2^ was 0.8453. Taken together, the above findings suggest that the study of TP has become a focus of attention and has entered a stage of rapid advancement.

### Contributions of countries/regions to global publications

We ranked the top 10 countries with the highest yields for all authors according to Np (**Table 1**). China published the most articles (306/20.94%), followed by India (233/15.92%) and America (137/9.36%). Papers from the United States were cited most frequently, with 3,244 citations, followed by papers from China (3,240) and India (2012). In addition, China had the highest H-index (30), followed by the United States (28). England, Taiwan, South Africa, and Egypt had similar Np, but the former three countries had significantly higher H-indices and Nc.

**Table 1:** Publications in the 10 most productive countries/regions.

Rank	Country/Region	Np	Nc	% (of 1464)	H-index
1	China	306	3240	20.90	30
2	India	233	2012	15.92	22
3	America	137	3244	9.36	28
4	South Korea	75	721	5.12	15
5	Japan	71	463	4.85	13
6	Egypt	67	151	4.58	6
7	England	67	1743	4.58	24
8	Taiwan	62	1189	4.23	20
9	South Africa	61	1938	4.17	27
10	Turkey	56	387	3.83	11

Np, number of publications; Nc, number of citations.

### Analysis of affiliations

**Table 2** shows the top 10 affiliations with the largest numbers of publications related to TP. EGYPTIAN KNOWLEDGE BANK has the highest Np (43), followed by SUN YAT SEN UNIVERSITY (35) and CAPITAL MEDICAL UNIVERSITY (33). Although the Np from the UNIVERSITY OF LONDON was 27, ranking fourth, its Nc (1,063) was the highest among the 10 most published units, and its H-index was also the highest (16). Although the EGYPTIAN KNOWLEDGE BANK had the highest Np, its Nc and H-index were far lower than those of the other affiliations. Among the top 10 most published affiliations, Chinese affiliations were the most common, accounting for three.

**Table 2:** The 10 most productive affiliations.

Rank	Affiliations	Np	Nc	Country	H-index
1	EGYPTIAN KNOWLEDGE BANK EKB	43	116	Egypt	5
2	SUN YAT SEN UNIVERSITY	35	543	China	14
3	CAPITAL MEDICAL UNIVERSITY	33	372	China	11
4	UNIVERSITY OF LONDON	27	1063	England	16
5	ALL INDIA INSTITUTE OF MEDICAL SCIENCES AIIMS NEW DELHI	26	633	India	13
6	HUAZHONG UNIVERSITY OF SCIENCE TECHNOLOGY	23	339	China	10
7	UNIVERSITY OF CAPE TOWN	23	999	England	15
8	STELLENBOSCH UNIVERSITY	22	721	South Africa	15
9	UNIVERSIDADE DE SAO PAULO	21	272	Brazil	10
10	POST GRADUATE INSTITUTE OF MEDICAL EDUCATION RESEARCH PGIMER CHANDIGARH	20	330	India	11

Np, number of publications; Nc, number of citations.

### Analysis of authors

**Table 3** shows the top 10 authors of published articles. In total, they published 164 papers, accounting for 11.20% of the total Np. Wu, CY from Sun Yat-Sen University ranked first in the field of TP in the number of published articles (23) and also had the highest H-index (13), followed by Lao, SH from Guangzhou Chest Hospital, China (19); Li, L (19) from Sun Yat-Sen University, China; and Shi, HZ (19) from Capital Medical University, China. Among the top 10 authors, Porcel JM from Spain had a very high Nc (653). It is worth noting that most of the top 10 authors were from China (7).

**Table 3:** The top 10 authors with the most publications.

Rank	Author	Country	Np	Nc	H-index
1	Wu CY	China	23	394	13
2	Lao SH	China	19	263	12
3	Li L	China	19	241	10
4	Shi HZ	China	19	241	10
5	Porcel JM	Spain	18	653	11
6	Antonangelo L	Brazil	15	232	9
7	Zhou Q	China	14	235	9
8	Chen XC	China	13	579	12
9	Zhang Y	China	13	150	7
10	Balboa L	Argentina	11	181	8

Np, number of publications; Nc, number of citations.

### Analysis of journals

As shown in **Table 4**, PLOS ONE had the largest Np on TP (58 publications, IF: 3.24). INTERNATIONAL JOURNAL OF TUBERCULOSIS AND LUNG DISEASE (44 publications, IF: 2.373) and BMC INFECTIOUS DISEASES (33 publications, IF: 3.09) ranked second and third, respectively. Approximately 20% of the papers were published in the top 10 academic journals (280/19.13%). PLOS ONE had the highest Nc (1297) and H-index (22). RESPIROLOGY had the highest impact factor of 6.424. All of the abovementioned journals are included in the SCI-expanded.

**Table 4:** The top 10 most active journals.

Rank	Journals	Np	Nc	IF(2020)	H-index
1	PLOS ONE	58	1297	3.240	22
2	INTERNATIONAL JOURNAL OF TUBERCULOSIS AND LUNG DISEASE	44	736	2.373	16
3	BMC INFECTIOUS DISEASES	35	453	3.090	13
4	MEDICINE	32	184	1.889	7
5	TUBERCULOSIS	24	263	3.131	9
6	RESPIROLOGY	23	605	6.424	13
7	JOURNAL OF CLINICAL MICROBIOLOGY	17	696	5.948	11
8	SCIENTIFIC REPORTS	17	217	4.380	7
9	INTERNATIONAL JOURNAL OF INFECTIOUS DISEASES	16	124	3.623	6
10	JOURNAL OF CLINICAL MICROBIOLOGY	17	696	5.948	11

Np, number of publications; Nc, number of citations; IF, impact factor.

It is worth noting that 4 journals that published numerous articles about TP were not listed in the SCI-expanded, namely, EGYPTIAN JOURNAL OF CHEST DISEASES AND TUBERCULOSIS (61), JOURNAL OF EVOLUTION OF MEDICAL AND DENTAL SCIENCES (31), CUREUS (19) and LUNG INDIA (18) (**Supplement Table 1**).

### Analysis of local citations

**Figure 4:**
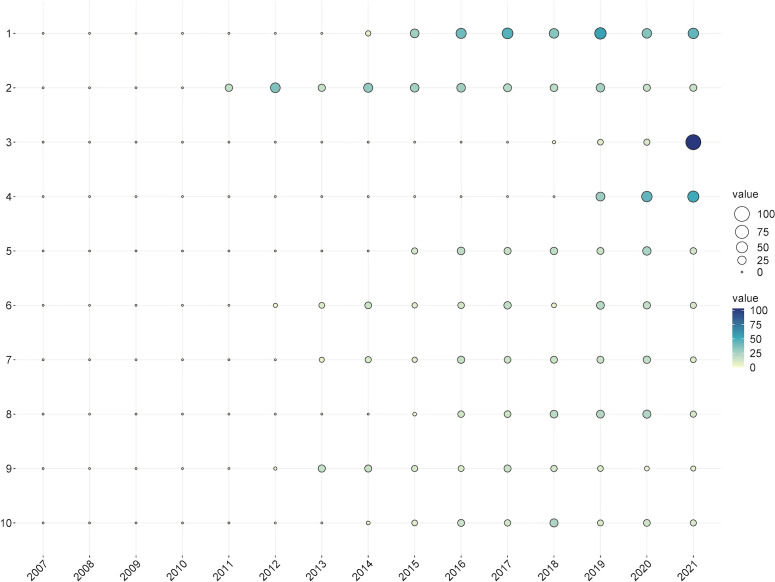
The yearly number of local citations of papers with high local citations (LCS). (The size and colors of the circle represent the LCS of papers).

The number of LCS per year for the top 10 articles is presented in **Figure 4**; **Table 5**. The number of LCS of the paper written by Denkinger (Denkinger et al., 2014) in 2014 was 291, ranking first. In this paper, the authors performed a systematic review and meta-analysis to assess the accuracy of the Xpert MTB/RIF assay for the detection of EPTB. In addition, the works of Hillemann et al. (Hillemann et al., 2011), (Kohli et al., 2018) and (Maynard-Smith et al., 2014) also provided some information about the diagnosis of EPTB using the Xpert assay, ranking 2^nd^, 4^th^, and 5^th,^ respectively. Light et al. (Light, 2011) and Vorster et al. (Vorster et al., 2015) reviewed the latest advances in TP care in detail at the time of publication, and the number of LCS of their works remained stable in recent years (6^th^ and 8^th,^ respectively). Li et al.’s work (3^rd^) on developing a new intelligent system for the diagnosis of TP had an increasing number of LCS over time (Li et al., 2018), while Jurado et al.’s work (9^th^) on the expression of IL-17 and interferon-gamma (INF-γ) in patients with active TB showed an opposite trend (Jurado et al., 2012). Regardless, these documents made a difference in the study of TP, as they prompted an increase the number of subsequent literature publications in this field.

**Table 5:** The top 10 cited articles.

Rank	Year	Article	IF(2020)	Total citations	Type of tudy
1	2014	Denkinger CM, et al. Xpert MTB/RIF assay for the diagnosis of extrapulmonary tuberculosis: a systematic review and meta-analysis. Eur Respir J. 2014 Aug;44(2):435-46.	16.671	291	Meta-Analysis
2	2011	Hillemann D, et al. Rapid molecular detection of extrapulmonary tuberculosis by the automated GeneXpert MTB/RIF system. J Clin Microbiol. 2011 Apr;49(4):1202-5.	5.948	256	Clinical Research
3	2018	Li C, et al. Developing a new intelligent system for the diagnosis of tuberculous pleural effusion. Comput Methods Programs Biomed. 2018 Jan; 153:211-225.	5.428	124	Clinical Research
4	2018	Kohli M, Schiller I, Dendukuri N, Dheda K, Denkinger CM, Schumacher SG, Steingart KR. Xpert^®^ MTB/RIF assay for extrapulmonary tuberculosis and rifampicin resistance. Cochrane Database Syst Rev. 2018 Aug 27;8(8):CD012768.	9.266	124	Meta-Analysis
5	2014	Maynard-Smith L, Larke N, Peters JA, Lawn SD. Diagnostic accuracy of the Xpert MTB/RIF assay for extrapulmonary and pulmonary tuberculosis when testing non-respiratory samples: a systematic review. BMC Infect Dis. 2014 Dec 31; 14:709.	3.090	118	Systemic Review
6	2011	Light RW, et al. Pleural effusions. Med Clin North Am. 2011 Nov;95(6):1055-70.	5.456	118	Review
7	2013	Critchley JA, et al. Corticosteroids for prevention of mortality in people with tuberculosis: a systematic review and meta-analysis. Lancet Infect Dis. 2013 Mar;13(3):223-37.	25.071	112	Meta-Analysis
8	2015	Vorster MJ, et al. Tuberculous pleural effusions: advances and controversies. J Thorac Dis. 2015 Jun;7(6):981-91.	2.895	104	Review
9	2012	Jurado JO, et al. IL-17 and IFN-γ expression in lymphocytes from patients with active tuberculosis correlates with the severity of the disease. J Leukoc Biol. 2012 Jun;91(6):991-1002.	4.962	103	Basic medical study
10	2014	Jiang J, et al. Mucosal-associated invariant T-cell function is modulated by programmed death-1 signaling in patients with active tuberculosis. Am J Respir Crit Care Med. 2014 Aug 1;190(3):329-39.	21.405	96	Basic medical study

IF, impact factor.

### Analysis of co-cited references

**Figure 5:**
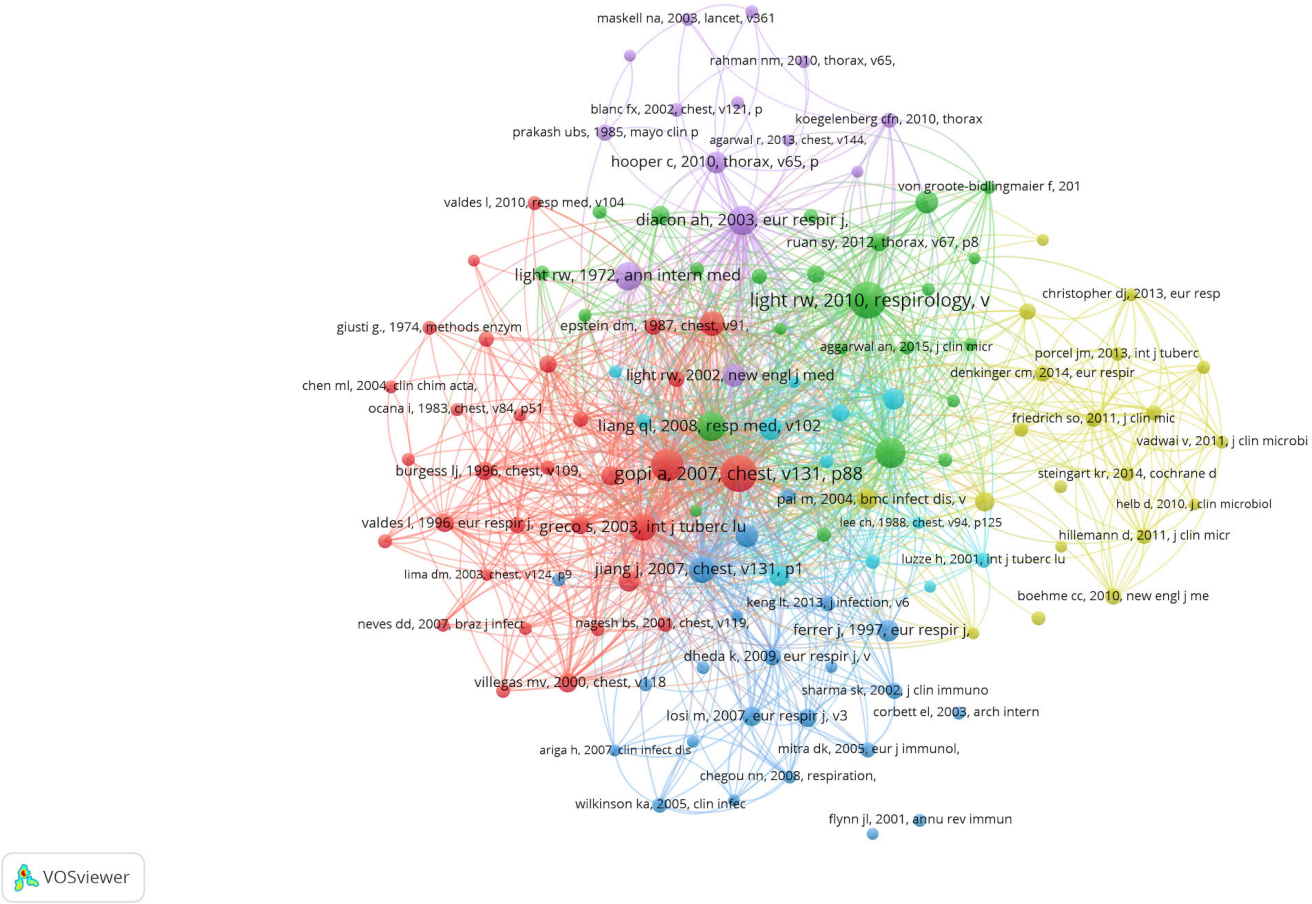
Mapping on co-cited references of studies related to tuberculous pleurisy (20 citations). Network map of co-cited references of studies related to tuberculous pleurisy. Of the 26, 449 references, 124 (classified into six clusters) had at least 20 times cited.

Unlike local citation analysis, cocitation network analyses emphasizes research themes closely related to a specific field. Considering the large number of cited references, the minimum number of reference citations was set to 20. Of the 26,449 references cited in the retrieved papers, 124 were selected for cocitation analysis (**Figure 5**). A line between two nodes indicates that both citations were cited in a single publication, and a shorter line represents a closer relationship between two papers. The size of the nodes represents the total link strength, representing the total number of cocitations of a document. In addition, different colored nodes were used to classify the papers into different clusters. Cluster 1 (in red) included 31 references that mainly focused on the diagnosis and treatment of TP. Cluster 2 (in green) mainly focused on immune-related biomarkers in diagnosis of the disease. Cluster 3 (in blue) focused on INF-γ. Cluster 4 (in yellow) focused on the Xpert assay. Cluster 5 (in purple) focused on pleural biopsy. Cluster 6 (in cyan) mostly focused on comorbid TB and HIV.

### Analysis of research hotspots

**Figure 6:**
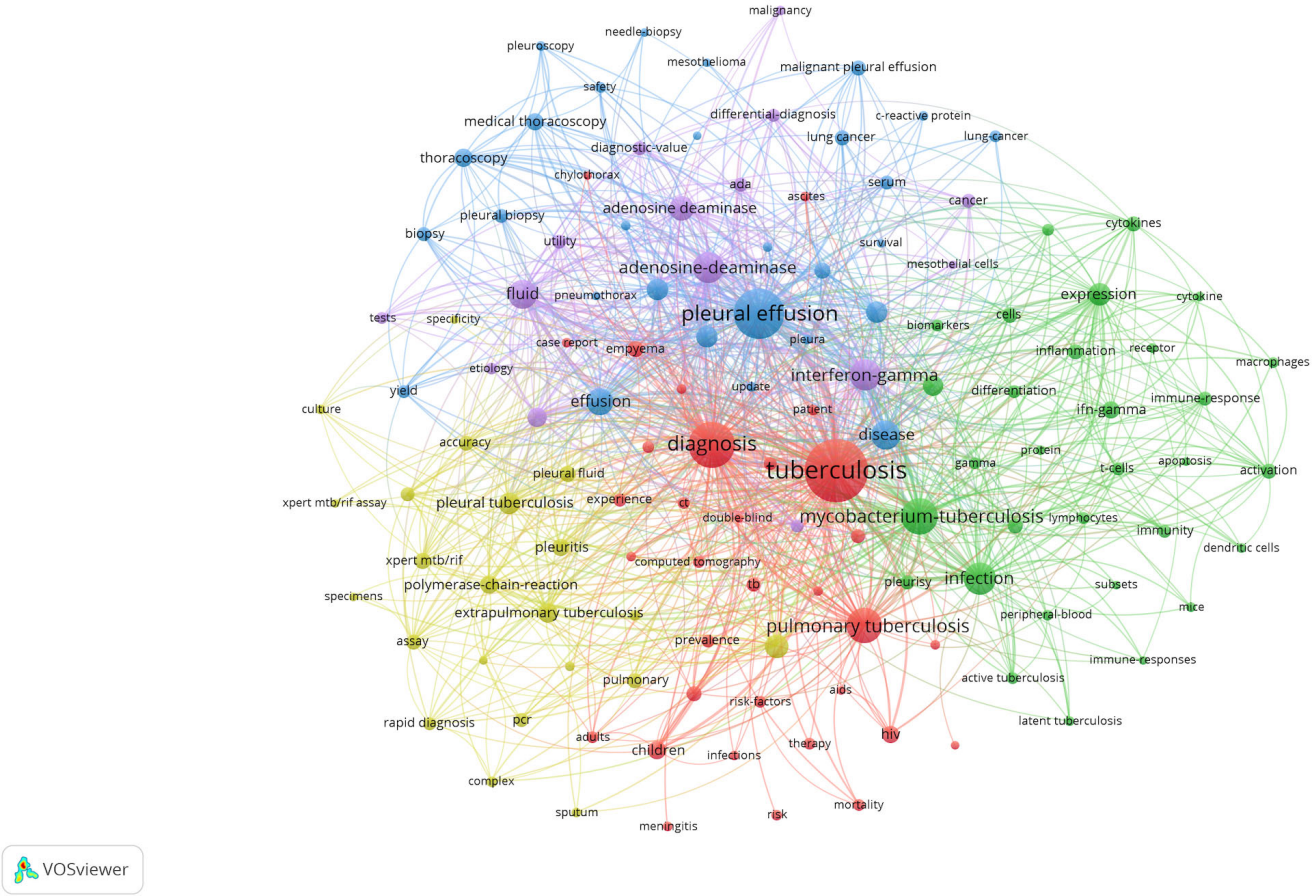
The mapping on keywords of tuberculous pleurisy. The 129 keywords that occurred more than 20 times were divided into five clusters by different colors: cluster 1: red, cluster 2: green, cluster 3: blue, cluster 4: yellow, cluster 5: purple. The size of the nodes represents the frequency of occurrences.

**Figure 7:**
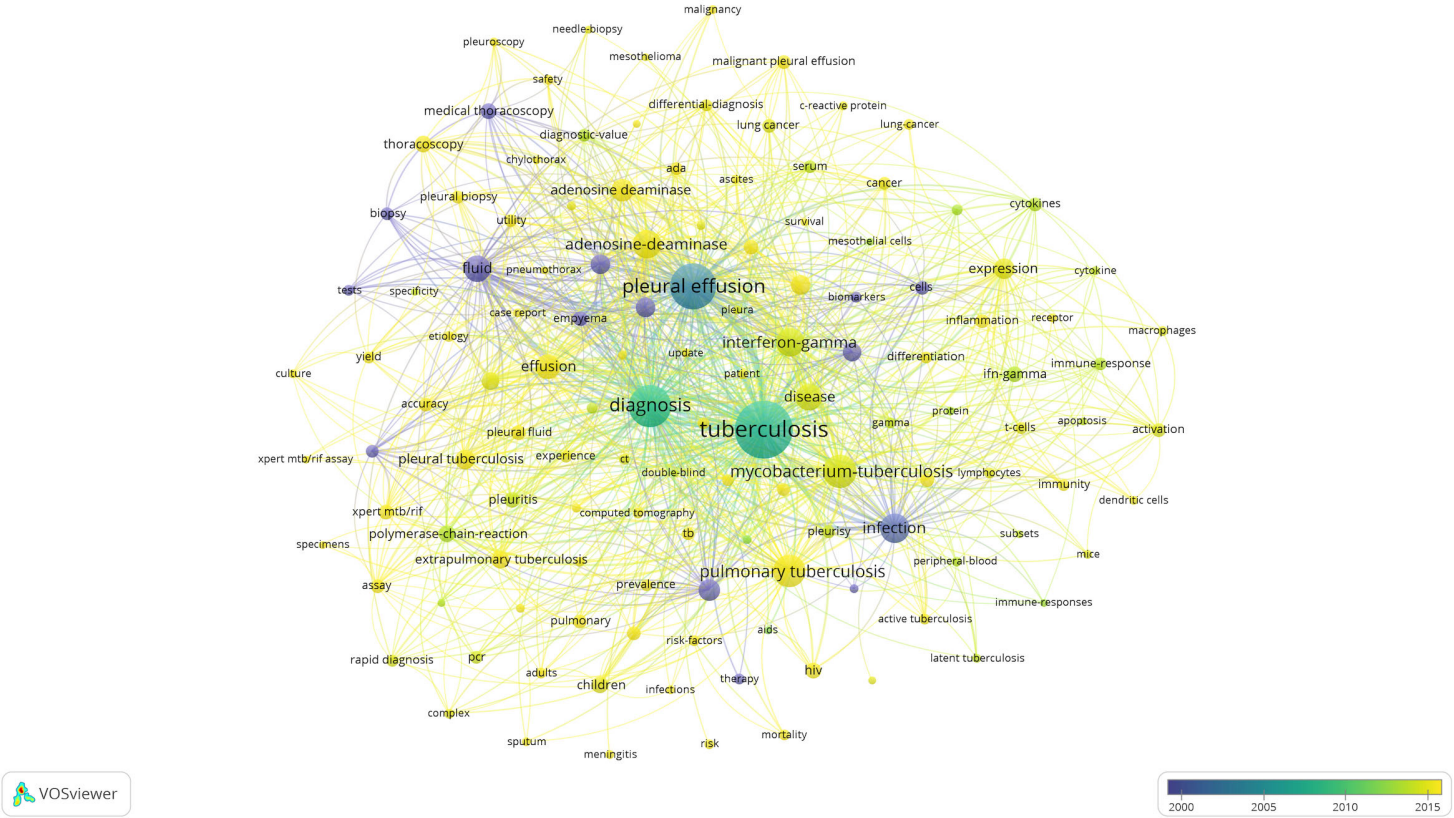
Visualization of keywords according to the APY. Keywords in yellow appeared later than that in blue.

In addition to search terms, keywords extracted from the titles and abstracts of 1,464 papers were analyzed by VOSviewer. According to **Figure 6**, cluster 1 mainly focused on the diagnosis of TP. Cluster 2 mainly focused on the effects of T cells and macrophages, as well as their cytokines. Cluster 3 focused on biopsy. Cluster 4 and cluster 5 mostly focused on the diagnostic value of rapid detection technologies, such as the Xpert assay in cluster 4 and biomarkers such as adenosine deaminase (ADA) and INF-γ release assays (IGRAs) in TP in cluster 5. The most frequently occurring keywords were “tuberculosis,” “diagnosis,” and “pleural effusion,” suggesting that the studies related to TP were mainly clinical studies that focused on diagnosis. As shown in **Figure 7**, the colors of all keywords were divided by VOSviewer according to the average publication year (APY). With research advancement over time, the research hotspots have changed from centralized to dispersed. During the first five years of the study period, the hotspots were ADA and IGRAs, while the latest hotspots were mainly genetic detection of *Mycobacterium tuberculosis* (MTB), including application of the Xpert MTB/RIF assay. However, studies on immune-related biomarkers of MTB have remained hotpots and are relatively limited.

## Discussion

In this study, we conducted a bibliometric analysis to investigate the developing trends and hotspots of research on TP from the SCI-expanded database by using VOSviewer and Bibliometrix software. We retrieved 1,464 original articles and reviews published from 2007 to 2021. According to the trend fitting curve, although there were fluctuations, the annual publication volume generally showed an upward trend, especially after 2014, and the annual publication volume has greatly increased over time. Groundbreaking articles with high LCS were the main drivers of annual publication growth.

Among the top-ranked countries, China ranked first, indicating that China is a high-volume country with regard to TP research. Moreover, among the top 10 units and authors, China had 3 units and 7 authors, which to a certain extent reflects the reasons for such high production in China. Interestingly, most of the top 10 countries were developing countries mostly in Asia and Africa, indicating the TP prevalence and burden in these countries. Although the Np in Asia was higher, the Nc and H-index were significantly lower than those in the United States. This was because many pioneering diagnostic techniques and diagnostic markers for TP were initially proposed by American scholars and the USA, such as Xpert (Park and Kon, 2021). And studies on TP were went deeper in this field as compared with the rest of the world (**Supplement Figure 1**). This also indicates that scholars and affiliations in Asia should make more efforts on the quality of their papers in this field to overcome the contradiction between the quantity and quality of publications.

Among the 10 SCI-expanded journals that had the largest Np on TP, 3 were relatively high-quality journals (IF>5.0). RESPIROLOGY had a higher publication volume and larger Nc, and the IF was 6.424, indicating that higher-quality journals pay close attention to studies on TP. It is worth noting that when not limited to SCI-expanded journals, 4 of the top 10 journals had no IF. Lack of an IF for two of these may be due to the regionality of Egypt and India, both of which have relatively high interest in TP, and for the other two may be due to the nature of the journals themselves (**Supplement Table 1**). It is also interesting to note that none of the articles in the top 10 LCS were published in the above journals. However, most of these articles were published in journals that had much higher IF, indicating that these studies were of high quality and more relevant to TP breakthroughs; this reminds researchers in this field to learn from these important studies. According to the contents of these studies, it was obvious that the diagnosis of TP by the Xpert MTB/RIF assay and immune-related biomarkers were critical research hotpots. According to the analysis of LCS, cocited references, and keywords, most of the research in this field was clinical research, and the research mainly focused on novel diagnostic techniques for TP.

As shown in **Figure 6**, the diagnosis of TP, specifically rapid diagnosis, has always been a research hotspot. TP diagnosis is often challenging because of the scarcity of bacilli in pleural fluid; the delayed growth of MTB, sometimes resulting in the need to conduct more invasive procedures to obtain pleural tissue for histological, microbiological or molecular examination (Antonangelo et al., 2019); the complicated etiology; the differential diagnosis of pleural effusion; and corresponding treatments. Thus, the rapid diagnosis of TP is of great significance. As shown in **Figures 7**, compared with pleural biopsy and thoracoscopy, the research topic that has receive the most attention in recent years has been the application of the Xpert assay in the diagnosis of TP. For the purpose of rapid diagnosis of TP, the Xpert assay was the method that researchers have paid the most attention to. The Xpert MTB/RIF assay detects TB DNA and can detect resistance to rifampicin directly from clinical specimens in approximately 2 hours, significantly improving the TB outcome by reducing the time to obtain results (Opota et al., 2019). In 2010, the WHO initially recommended Xpert MTB/RIF for test of TB. However, Xpert presented less-sensitive features for specimens with low bacterial loads. The WHO published updated guidance on use of Xpert in 2013. This updated policy statement expanded recommendations for use of Xpert for pulmonary TB in adults and provided additional guidance on use of the test for childhood TB and EPTB (Kohli et al., 2018). In 2017, the WHO endorsed the new-generation Xpert technology, named Xpert MTB/RIF Ultra (Xpert-Ultra), to replace Xpert for HIV patients, children, or EPTB (Park and Kon, 2021). Several studies found that when pleural fluid was used to diagnose TP, the Xpert assay had high specificity (100%) for the diagnosis of TP-related pleural effusion, indicating its value in confirming a TP diagnosis; however, the low sensitivity (14–34%) indicates that the Xpert assay is of limited value for TP screening (Maynard-Smith et al., 2014; Huo and Peng, 2018; Li et al., 2020). Low sensitivity may be attributed to the presence of Polymerase Chain Reaction (PCR) inhibitors in pleural fluid, or the loading of MTB is too low that the detection limit cannot be reached even by centrifugation (Huo and Peng, 2018). Several studies have demonstrated insufficient diagnostic accuracy of the Xpert MTB/RIF assay in oligobacterial disease (especially in HIV-coinfected patients or children), thus limiting its use for TP (Nicol et al., 2011; Theron et al., 2011; Sohn et al., 2014). The Xpert Ultra assay was developed to overcome the shortcomings of the Xpert MTB/RIF assay but appears to have had little success in the diagnosis of TP (Wu et al., 2019). According to the abovementioned evidence, the Xpert assays have variable but low sensitivity. Therefore, to advance the application of the Xpert assay in the diagnosis of TP, we should increase the efficiency of DNA amplification to improve the sensitivity of this technology in the diagnosis of TP. Recent research has shown that the role of the Xpert assay in the diagnosis of TP has not improved much over time, and its limitations suggest that this technology is not valuable for screening and diagnosis in areas with a high TB incidence. While, based on the rapidity and high specificity of its detection, there have been studies using it as an indicator to predict the prognosis of TB patients with HIV (Feasey et al., 2013) or treatment outcomes (Pires et al., 2020).

Immune-related biomarkers for the diagnosis of TP have consistently remained research hotspots and play an irreplaceable role in the diagnosis of TP; the diagnostic performance of biomarkers is much better than that of the Xpert assay. Lymphocyte recruitment is an important process in the pathological process of TP. Lymphocyte recruitment leads to an intense T-helper type 1 (TH1) cell-mediated delayed hypersensitivity reaction to MTB. The TH1-driven immune response releases ADA and INF-γ, which are important in the diagnosis of TP.

A meta-analysis of 16 studies including over 4,000 patients revealed that the ADA level had a sensitivity and specificity for the diagnosis of TP of approximately 90%, irrespective of the cutoff used (it varied between 23 and 45 U/L in studies) (Palma et al., 2019). Similar to the above study, two Chinese cohort studies from Beijing and Wuhan, with 154 and 120 patients, respectively, reported sensitivities of 88–89% and specificities of 86–87% for ADA, with a cutoff of 21.4 U/L (Wang et al., 2018). In the most comprehensive meta-analysis to date, Aggarwal et al. showed that pleural fluid ADA has a high sensitivity of 0.92 and specificity of 0.9 for the diagnosis of TP (Aggarwal et al., 2019). These studies demonstrate that the ADA level is an effective indicator for the diagnosis of TP. However, we still need to pay attention to detection in the immunosuppressed population. In addition, the optimal cutoff value for ADA requires more research to determine the optimal sensitivity and specificity values.

IGRAs quantify IFN-γ released by T-lymphocytes in response to stimulation by specific antigens encoded in region of difference 1 (RD1) of the MTBgenome (Aggarwal et al., 2015). Previous studies have fully verified the high diagnostic performance of the T-SPOT test (an IGRA) in the diagnosis of TP. A recent meta-analysis (Luo et al., 2020a) showed the following pooled values for the diagnostic accuracy of the PF T-SPOT test: sensitivity, 0.91% (95% CI, 0.89%-0.92%, I^2^ = 80.9%); and specificity, 0.88% (95% CI, 0.86%-0.91%, I^2^ = 87.3%). Other studies reported that the sensitivity and specificity of the PF T-SPOT test for the diagnosis of TP were 89.76% -91.07% and 94.90% - 96.70%, respectively (Luo et al., 2019; Luo et al., 2020b). The diagnostic accuracy of the PF T-SPOT assay was better than that of other routine tests, such as pathogen detection methods and biochemical marker detection. Furthermore, in a meta-analysis of 14 studies involving 932 patients with TB effusions, IGRAs (either the T-SPOT or QuantiFERON test) displayed a pooled sensitivity and specificity of only 72% and 78%, respectively (Aggarwal et al., 2015). Another meta-analysis revealed that the sensitivity and specificity of IGRAs for the detection of pleural effusion in the diagnosis of TP were 72~95% and 70~92.19%, respectively (Tong et al., 2021). One of the included articles indicated that IGRAs have better diagnostic performance than pleural biopsy for the diagnosis of pleural effusion (Cirak et al., 2012). A recent study showed that the INF-γ ultrasensitive rapid immunosuspension assay (IRISA-TB) demonstrated a higher specificity and rule-in value than ADA in a high-TB-burden setting where HIV is endemic (Meldau et al., 2019). The differences in the diagnostic performances of IGRAs in different studies have not been well analyzed, but the diagnostic performance of the T-SPOT test is higher than those of the other tests. However, the operation of the T-SPOT test is complicated, so future research should focus on simplification, downscaling and the portability of this technology. Since IGRAs were originally designed to use peripheral blood samples, the interpretation criteria have not been validated for use on pleural fluid, indicating that more studies should be conducted to verify the applicability.

The above immune-related indicators have corresponding commercial detection products; however, some researchers are committed to the exploration of new immune-related indicators that can effectively diagnose TP, among which interleukin-27 (IL-27) is a noteworthy indicator. Shi et al. indicated that IL-27 in pleural fluid is a sensitive and specific biomarker for the differential diagnosis of TB pleural effusion, differentiating TB plural effusion from pleural effusion due to other causes (Yang et al., 2012). Wang et al. reported that IL-27 detection in plural fluid demonstrated a sensitivity and specificity of 96% and 99%, respectively, using a cutoff value of 591.4 ng/L (cutoff 116 ng/L: sensitivity 92%, specificity 95%) (Wang et al., 2018). A meta-analysis of pleural fluid IL-27 levels in 550 patients with TP or malignant pleural effusion (MPE) confirmed the above findings (pooled sensitivity was 93%, pooled specificity was 97%) (Liu et al., 2018). However, the influence of tumor type on the detection of IL-27, especially IL-27-related tumors (Horlad et al., 2016), could not be determined, so relevant studies are needed for verification. At the same time, the lack of general availability and relatively long turnaround time of cytokine testing limit the use of IL-27 for the diagnosis of TP. Furthermore, no optimal cutoff value has uniformly been accepted, the reported ones ranging from 391 ng/L to 1,007 ng/L (Yang et al., 2012; Skouras et al., 2015).

Through the combination of various biomarkers, we are committed to improving the sensitivity and specificity of diagnostic methods for TP. Numerous clinical studies and meta-analyses were performed to compare the diagnostic yields of different combinations of diagnostic methods. One meta-analysis (Tong et al., 2021) indicated that PF-IGRA combined with ADA detection could increase the diagnostic accuracy of TP. A recent study (Fenhua et al., 2021) showed that the specificity and sensitivity combined pleural effusion IL-33 detection, ADA detection and the peripheral blood T-SPOT test were 100% and 88.5%, respectively. A study by Zhang et al. showed that the relative expression levels of INF-γ mRNA and IL-27 mRNA in the TP group were significantly higher than those in the control group (p<0.05), indicating that the combination of the T-SPOT test with INF-γ and IL-27 detection has significant value in the clinical diagnosis of TP. All these studies clearly demonstrated that combined detection of pleural effusion can improve the diagnostic efficacy of TP, providing researchers with future research directions. However, simplifying operations and improving efficiency to obtain a rapid diagnosis and reduce costs are issues that need to be considered.

As mentioned above, most of the recent research on TP has focused on the diagnosis of TP, and there were few treatment-related studies. This may be related to the lack of studies on the difficulty of isolating MTB from TB pleural effusion fluid for drug-resistance analysis. Therefore, how to integrate diagnosis and drug resistance testing should be addressed. In this regard, the Xpert MTB/RIF and Xpert Ultra assays have advantages, but as noted in the above studies, the relatively low sensitivity limits their use for the diagnosis of TP. With the continuous development of sequencing technology, relevant studies have applied third-generation sequencing technology for the detection of MTB. A recent study by Harriet D et al. (Gliddon et al., 2021) showed that the sensitivity and specificity of rifampin resistance prediction by targeted isothermal amplification-nanopore sequencing were 96.3% (95% confidence interval [CI], 81.0 to 99.9%) and 100.0% (95% CI, 15.8 to 100.0%), respectively. For isoniazid resistance prediction, the sensitivity and specificity were 100.0% (95% CI, 86.3 to 100.0%) and 100.0% (95% CI, 39.8 to 100.0%), respectively; this allowed the diagnosis of drug-resistant TB in a shorter time, with less equipment, and for a lower price than current methods. Similar studies (Smith et al., 2020) have also shown Nanopore’s competitiveness in the diagnosis of drug-resistant TB. A recent review (Dippenaar et al., 2022) showed that Nanopore’s low cost of sequencing combined with its portability and good performance makes it an attractive option for sequencing in research and clinical care settings. However, data on its application in the field of TB are limited, and there is no research on its application in the diagnosis of TP. However, the technology has been applied to detect drug-resistant TB infection. Therefore, future research may focus on the application of new sequencing technologies for TP diagnosis that can integrate TP diagnosis and drug-resistance detection.

A definitive diagnosis of TP can only be confirmed by identification of MTB by microscopy and/or culture in sputum, pleural fluid, or pleural biopsy specimens. Unfortunately, it can only be achieved in relatively few cases. In one study, acid-fast smears and solid culture media of sputum samples were positive in 8% and 41%, respectively (Valdés et al., 2012). In the absence of an adequate sputum sample, sputum must be induced by inhalation of saline. In another previous study, only 6% and 36% of pleural fluid smears and cultures were positive. At the same time, the yields of examining acid-fast bacilli and cultured pleural biopsy were 24% and 53%, respectively (Sahn et al., 2013). Meanwhile, the isolation time of MTB is long, and it may take 4-6 weeks for clinical specimens to become positive when cultured in solid medium, which prevents immediate clinical decision-making and timely treatment. Therefore, the diagnosis of TP is difficult and often based on clinical empirical treatment and judgment. There is an urgent need for more sensitive and specific diagnostic methods to help improve the diagnosis of TP. Consequently, research related to TP diagnosis has exploded in recent years.

In general, it was interesting that review articles get more citations than primary research articles. On the one hand, because the diagnosis and treatment of TP are not yet clear, especially in the field of diagnosis, while the innovation of diagnosis technology and diagnosis-related markers is developing rapidly. Therefore, original articles often only focus on one research point. As a scientific researcher, it is difficult to track the progress of disciplines outside the field. Researchers seek to quickly understand the need for important advances in the field. A review is a collection of literature works, and its overview of important progress and original work greatly saves researchers the effort and time to find and interpret a large number of original articles. On the other hand, review articles with high citation rates are mainly systematic reviews and meta-analyses. Such articles often focus on a specific small issue for detailed analysis and summary, and have high level of evidence which can often give readers a better understanding of the progress and role of a specific issue.

Based on the bibliometric analysis and bibliographic visualization, the development trends and hotspots in this field can be understood to a certain extent. Our research used LCS as an indicator, which may help to better understand important nodes in the trends in this field. However, this study has some limitations. First, only English articles and reviews from the SCI-expanded were included. Second, VOSviewer may miss some information because it cannot analyze the full texts of the publications. Finally, due to the exclusion of some excellent newly published papers with low Ncs, this study has a certain degree of lag.

## Conclusion

The bibliometric analysis showed that recent research on TP has evolved rapidly. China and other Asian countries were major producers, and the United States has made many outstanding breakthroughs in this field. Recently, the diagnosis of TP, especially the rapid diagnosis of TP, has become a research hotspot. Moreover, immune-related biomarkers remain extremely important in TP diagnosis. Additional studies should emphasize the combination of different biomarkers to improve the sensitivity and specificity of TP diagnosis. Overall, the development of novel diagnosis technologies is significant and can allow TP to be diagnosed in a rapid, economical and convenient manner.

## Data availability statement

The original contributions presented in the study are included in the article/**Supplementary Material**. Further inquiries can be directed to the corresponding author.

## Author contributions

YB and MD did the bibiometric analysis and contributed to manuscript writing. QZ participated in experimental design and manuscript writing. GH designed this study and organized the manuscript writing. All authors contributed to the article and approved the submitted version.

## Funding

This research was supported by National High Level Hospital Clinical Research Funding (2022-NHLHCRF-LX-01), the Elite Medical Professionals project of China-Japan Friendship Hospital (No. ZRJY2021- BJ08), and the Nonprofit Central Research Institute Fund of Chinese Academy of Medical Sciences (No. 2020-PT320-001).

## Conflict of interest

The authors declare that the research was conducted in the absence of any commercial or financial relationships that could be construed as a potential conflict of interest.

## Publisher’s note

All claims expressed in this article are solely those of the authors and do not necessarily represent those of their affiliated organizations, or those of the publisher, the editors and the reviewers. Any product that may be evaluated in this article, or claim that may be made by its manufacturer, is not guaranteed or endorsed by the publisher.
