# Supplementary material for: Global trends of research on tuberculous pleurisy over the past 15 years: A bibliometric analysis
Source: Front Cell Infect Microbiol. 2022 Aug 30;12:937811. doi: 10.3389/fcimb.2022.937811 (PMC9468418; doi:10.3389/fcimb.2022.937811)
Supplement: Supplementary file 1 [file DataSheet_1.docx]

Supplementary Material

**Supplementary table 1 The 10 most active journals (including journals removed from SCI)**

| Rank | Journals | Np | Nc | IF (2020) | H-index |
| --- | --- | --- | --- | --- | --- |
| 1 | EGYPTIAN JOURNAL OF CHEST DISEASES AND TUBERCULOSIS | 61 | 102 | Removed | 5 |
| 2 | PLOS ONE | 58 | 1297 | 3.24 | 22 |
| 3 | INTERNATIONAL JOURNAL OF TUBERCULOSIS AND LUNG DISEASE | 44 | 736 | 2.373 | 16 |
| 4 | BMC INFECTIOUS DISEASES | 35 | 453 | 3.09 | 13 |
| 5 | MEDICINE | 32 | 184 | 1.889 | 7 |
| 6 | JOURNAL OF EVOLUTION OF MEDICAL AND DENTAL SCIENCES JEMDS | 31 | 7 | Removed | 2 |
| 7 | TUBERCULOSIS | 24 | 263 | 3.131 | 9 |
| 8 | RESPIROLOGY | 23 | 605 | 6.424 | 13 |
| 9 | CUREUS | 19 | 15 | Removed | 2 |
| 10 | LUNG INDIA | 18 | 71 | Removed | 5 |

**Abbreviations:** Np, number of publications; Nc, number of citations; IF, impact factor.

**
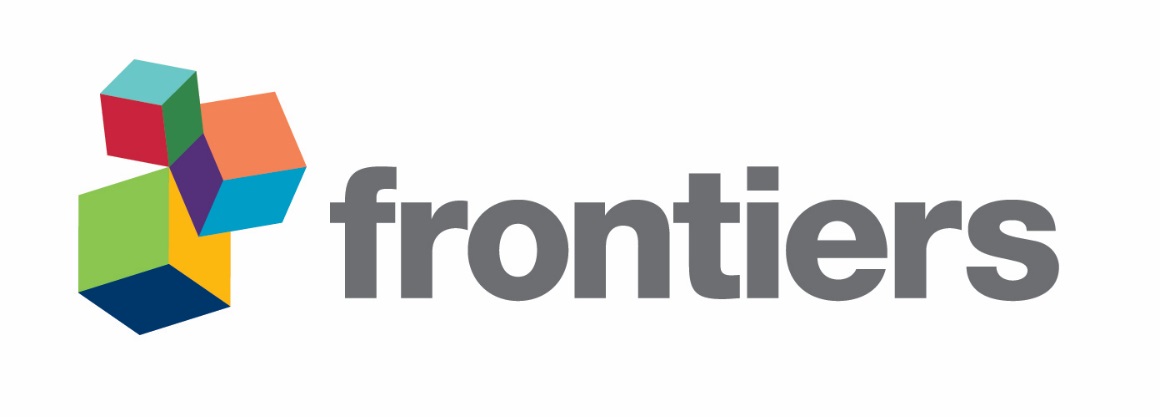
**

**Supplementary Figure 1.** **Number of studies about Xpert in different countries**

**
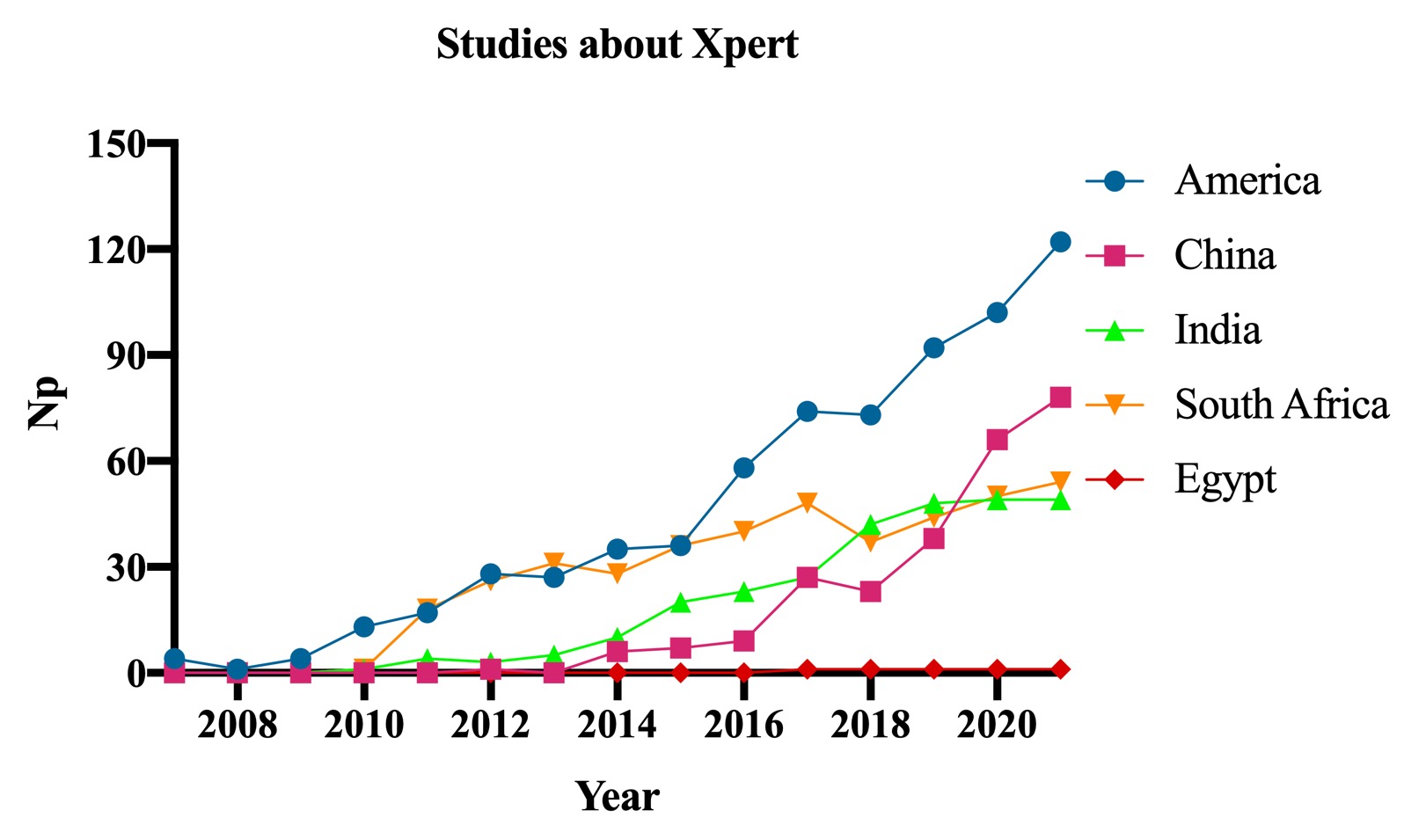
**
